# Unhealthy weight among children in Spain and the role of the home environment

**DOI:** 10.1186/s13104-018-3665-2

**Published:** 2018-08-15

**Authors:** Elizabeth Vaquera, Rebecca Jones, Pau Marí-Klose, Marga Marí-Klose, Solveig Argeseanu Cunningham

**Affiliations:** 1 Department of Sociology and Trachtenberg School of Public Policy and Public Administration, Cisneros Hispanic Leadership Institute, The George Washington University, 2114 G Street NW, Washington, DC 20052 USA; 2 Nutrition and Health Sciences, Emory University, 1518 Clifton Rd NE, Atlanta, GA 30322 USA; 3 Facultad de Ciencias Sociales y del Trabajo, Universidad de Zaragoza, Saragossa, Spain; 4 Dept. Sociologia i Anàlisi de les Organitzacions, Universitat de Barcelona, Barcelona, Spain; 5 Hubert Department of Global Health, Emory University, Atlanta, USA

**Keywords:** Obesity, Overweight, Underweight, Family, Spain

## Abstract

**Objective:**

Unhealthy weight is a major global health concern. This study examines unhealthy weight among children in Spain and the role of the home environment therein. Data are from a 2010 national survey of families with children. We examined unhealthy weight among children ages 5–10 years using the WHO Child Growth Standards and used multivariate logistic regression to assess associations with family characteristics.

**Results:**

There was a high prevalence of unhealthy weight, with only 46% of children at normal weight. Both underweight and obesity were higher among boys (14%; 22%) than girls (13%; 12%). Underweight and obesity were higher among children of mothers with obesity and those with unemployed parents. Obesity was higher among children of mothers who were less educated (35%) and among children of immigrants (19%). We find high levels of unhealthy weight in children, with both underweight and obesity being predicted by the same family environment characteristics.

**Electronic supplementary material:**

The online version of this article (10.1186/s13104-018-3665-2) contains supplementary material, which is available to authorized users.

## Introduction

Underweight and obesity each contribute to negative health outcomes [1]. Obesity is a factor in morbidity and mortality from chronic conditions such as diabetes and cardiovascular disease globally [2]. Obesity during childhood is a major health concern due to its growing prevalence and the short- and long-term health problems [3–5]. Underweight is known to exacerbate morbidity and mortality from infectious conditions such as tuberculosis and malaria [2].

## Main text

### Background

Spain experienced an economic crisis starting in 2008, followed by a slow recovery [6], leading to concerns about healthy growth in children [7]. According to the Childhood Obesity Surveillance Initiative (COSI) (2008–2009), Spain has the second highest prevalence of obesity in Europe among primary school age children (22% in children age 9 years), just behind Greece (31%) [5, 8]. Underweight among school-aged children in Europe is estimated to range between 3.3% in Greece [9] to 11.7% in Lithuania [10, 11]. Disparities in unhealthy weight across Europe have been attributed to differences in consumption patterns, levels of physical activity and inactivity, and childcare practices [12].

Children’s weight is thought to be heavily influenced by family environment through quality and quantity of food, eating patterns, and distribution of resources among family members [13–15]. The family environment may be linked with child nutrition through child’s age, gender, and daycare attendance; mother’s age at childbirth; parents’ weight, marital status, employment, and education; co-residence of grandparents and siblings; family income and activities [14–31]. The associations between these factors and weight sometimes differ across countries; for example, parents’ marital status has been associated with obesity in the US [18], but not in Norway [19].

In Spain, children whose parents have obesity have higher odds of overweight [23]. Parents’ socioeconomic status and employment, particularly mothers’ employment, are associated with higher obesity risks among children and youth [25, 28], though these associations have not been observed in other European countries [19, 26, 27]. Even in higher-income countries, economic crises can lead to malnutrition, both underweight [7] and obesity [32].

Previous studies of unhealthy weight in Europe have focused on obesity [8], but do not examine under-nutrition [10]. In light of recent economic crises in high-income countries, including Spain, it is important to consider both underweight and overweight. We present estimates of the prevalence of unhealthy weight—underweight, overweight, and obesity—among elementary school-aged children in Spain and explore associations with the home environment.

### Data

Data are from *Encuesta de Relaciones Inter e Intrageneracionales en la Infancia,* a nationally representative survey of households with children ages 0–10 years in Spain [33] fielded in 2010 (n = 2206). The response rate was 33.4%, which is similar to published studies employing these methods [34]. Sampling was done through a proportionate-to-population cluster design using regions as clusters and households with children within regions stratified by size of the municipality, child’s sex and age. Interviews were conducted over the phone in Spanish using computer-assisted telephone interviewing (CATI) software. Data were collected with the previous verbal informed consent of the participants and researchers had only access to deidentified data, guaranteeing their anonymity and privacy. According to ethical assessment in Spain [35], no ethical assessment was necessary. The present study uses data from interviews with the parents of school-aged children (ages 5–10 years) who participated in a 62-item parent questionnaire (n = 844) (Additional file 1). Many questions draw from well-established surveys of child wellbeing such as ECLS-K, Add Health, or The Fragile Families and Child Wellbeing Study [35–38].

### Methods

To measure unhealthy weight, we evaluated children’s weight status using the WHO growth reference for school-aged children, which estimates BMI z-scores relating a child’s weight and height to children of the same age and sex from a reference population. Based on parent-reported weight, height, sex, and age of children, we constructed BMI z-scores and used the WHO cut-off points for underweight (< −2 SD below the mean), normal weight (≥ −2 and < 1), overweight (≥ 1 and ≤ 2 SD above the mean), and obesity (≥ 2 SD above the mean) [39].

We examined characteristics that may be linked with unhealthy weight based on previous literature. *Bio*-*demographic* measures included child’s gender and age, whether the parents reported the child to be in good health, mother and father’s BMI, and mother’s age at the child’s birth. The parent who answered the questionnaire reported their own and their partner’s height and weight. Child overall health question was recorded as good, fair, or poor. *Family structure* measures included whether the parents were married; whether any grandparent was living in the home; and whether the child had siblings living in the home. F*amily activities* measures included which meals (breakfast, lunch, dinner) were eaten with the family, whether the child engaged in active pastimes daily outside of school, whether the child watched television or used a computer or video games daily; and whether the child came home for lunch on schooldays—in Spain all schools and many employers provide a 2 to 3-h lunch recess so families can eat at home together if they wish. *Socio*-*economic* measures included monthly household income, whether the mother and father were employed, whether the mother had completed more than primary education, whether at least one parent was born in another country. Immigrants tend to have higher BMIs with increasing stay in a new destination country [29]. We also considered whether the child had attended day care before age 3, which has been associated with differences in weight status [14].

After diagnostic tests of distributions and multicollinearity, prevalence of underweight, normal weight, overweight, and obesity were calculated. We calculated the distribution of each characteristic among children experiencing underweight, normal-weight, overweight or obesity, testing for significant differences using t-tests. We then estimated multinomial logistic regression, with the outcome categories being underweight, normal weight (reference), overweight, and obesity. STATA 11.1 was used for all analyses with significance based upon 95% confidence intervals.

### Results

More than half of children ages 5–10 years old had unhealthy weight (54%): 14% underweight, 24% overweight, and 17% obesity (Table 1). Boys were more often in unhealthy weight categories: 61% of boys were of unhealthy weight (14% with underweight, 25% with overweight and 22% with obesity) compared with 47% of girls (13% with underweight, 23% with overweight and 12% with obesity); the difference was largely driven by the higher prevalence of obesity among boys.

**Table 1:** Characteristics of Children (5–10 years) from nationally representative *Encuesta de Relaciones Inter e Intrageneracionales en la Infancia* (2010) in Spain (n = 844)

	Mean or proportion	95% CI
Bio-demographic characteristics of child		
Weight status		
Underweight	0.14	(0.12, 0.17)
Normal weight	0.46	(0.43, 0.48)
Overweight	0.24	(0.22, 0.26)
Obese	0.17	(0.15, 0.19)
Boy	0.50	(0.46, 0.53)
Age (years)	7.62	(7.51, 7.73)
Good health	0.95	(0.94, 0.97)
Parent’s BMI		
Mother	23.59	(23.33, 23.85)
Father	26.30	(26.06, 26.54)
Mother’s age at childbirth	33.78	(33.45, 34.11)
Household structure		
Married parents	0.87	(0.85, 0.89)
Has at least one sibling	0.71	(0.68, 0.74)
Co-resides with a grandparent	0.08	(0.06, 0.10)
Family activities		
Family eats all 3 meals together	0.13	(0.10, 0.15)
Plays sports/does physical activity daily	0.49	(0.46, 0.53)
Daily screen time	0.20	(0.17, 0.22)
Family socio-economic characteristics		
Unemployed mother	0.37	(0.33, 0.40)
Unemployed father	0.10	(0.08, 0.13)
Mother with primary or less education	0.26	(0.23, 0.29)
At least one foreign-born parent	0.14	(0.11, 0.16)
Child attended daycare before age 3 years	0.64	(0.60, 0.67)
Monthly household income		
€1200 or less	0.17	(0.13, 0.19)
€1201–€2000	0.33	(0.31, 0.35)
€2001–€3000	0.32	(0.29, 0.35)
€3001 or more	0.19	(0.17, 0.21)

95% confidence intervals are in parentheses

Weight status was defined using World Health Organization child growth reference standards. Underweight defined as BMI-for-age-z-score < − 2. Normal weight defined as BMI-for-age-z-score ≥ − 2 and < 1. Overweight defined as BMI-for-age-z-score ≥ 1 and ≤ 2. Obese defined as BMI-for-age-z-score defined as ≥ 2

Good health was parent’s report of child’s overall health on a scale of good, fair or poor

Daily screen time was parent reported if the child watched television or used a computer or video games daily

Mother with primary or less education was defined as whether the mother had completed primary school education or less

Data source: *Encuesta de Relaciones Inter e Intrageneracionales en la Infancia (2010)*

Children were on average 8 years old. Almost 90% of children have married parents, 71% have at least one sibling, and 8% live with at least one grandparent. Fourteen percent of children had at least one foreign-born parent. Sixty-four percent had attended day-care before the age of 3 years. Thirteen percent ate all three meals at home daily, about half did some physical activity every day, and 20% had screen time daily. A quarter of mothers had no more than primary education and over a quarter of mothers (37%) and 10% of fathers were not working at the time of the interview, consistent with the high unemployment levels in Spain at the time.

The average mother had normal weight (BMI = 23.6), while the average father was slightly overweight (BMI = 26.3). Only 27% of children had two parents with normal weight, 5% had at least one underweight parent (in 80% of cases the mother), and 25% had at least one parent with obesity (in 70% of cases, the father).

Children experiencing overweight or obesity were more often boys (Table 2); they more often had parents with overweight or obesity and mothers with only primary education; they less frequently had attended daycare before the first 3 years of life.

**Table 2:** Characteristics of family and children (5–10 years) from *Encuesta de Relaciones Inter e Intrageneracionales en la Infancia (2010)* by weight category (n = 844)

	Underweight		Normal weight		Overweight		Obesity		Significant differences between weight categories
	n = 115		n = 389		n = 200		n = 140		
	Mean or proportion	95% CI	Mean or proportion	95% CI	Mean or proportion	95% CI	Mean or proportion	95% CI	
Bio-demographics									
Boy	0.52	(0.43, 0.62)	0.42	(0.37, 0.47)	0.52	(0.45, 0.59)	0.65	(0.57, 0.73)	c, d, e, f
Age (years)	7.56	(7.26, 7.86)	7.67	(7.51, 7.84)	7.75	(7.53, 7.97)	7.35	(7.07, 7.63)	f
Good health	0.94	(0.90, 0.98)	0.96	(0.95, 0.98)	0.96	(0.93, 0.98)	0.93	(0.89, 0.97)	
Parent’s BMI									
Mother	22.61	(22.12, 23.10)	23.51	(23.14, 23.89)	23.88	(23.37, 24.39)	24.51	(23.78, 25.24)	a, b, c, e
Father	25.44	(24.93, 25.94)	26.08	(25.73, 26.42)	26.60	(26.07, 27.13)	27.23	(26.65, 27.81)	a, b, c, e
Mother’s age at childbirth	33.46	(33.39, 35.23)	33.65	(33.18, 34.)	33.59	(32.91, 34.27)	33.99	(33.11, 34.87)	
Household structure									
Married parents	0.87	(0.81, 0.93)	0.88	(0.85, 0.92)	0.85	(0.79, 0.90)	0.86	(0.80, 0.92)	
Has at least one sibling	0.72	(0.64, 0.80)	0.73	(0.69, 0.77)	0.69	(0.63, 0.75)	0.70	(0.62, 0.78)	
Co-resides with a grandparent	0.07	(0.02, 0.12)	0.08	(0.05, 0.10)	0.07	(0.03, 0.11)	0.13	(0.07, 0.18)	
Family activities									
Family eats all 3 meals together	0.08	(0.03, 0.13)	0.12	(0.08, 0.15)	0.16	(0.10, 0.21)	0.16	(0.10, 0.22)	b, c
Plays sports/does physical activity daily	0.41	(0.32, 0.50)	0.54	(0.49, 0.59)	0.47	(0.40, 0.53)	0.49	(0.40, 0.57)	a
Daily screen time	0.18	(0.11, 0.25)	0.19	(0.15, 0.22)	0.21	(0.15, 0.27)	0.23	(0.16, 0.30)	
Family socio-economic characteristics									
Unemployed mother	0.30	(0.21, 0.38)	0.36	(0.31, 0.41)	0.40	(0.33, 0.46)	0.41	(0.33, 0.49)	
Unemployed father	0.04	(0.001, 0.07)	0.11	(0.08, 0.14)	0.11	(0.06, 0.15)	0.14	(0.08, 0.20)	a, b, c
Mother with primary or less education	0.24	(0.16, 0.32)	0.24	(0.19, 28)	0.24	(0.18, 30)	0.35	(0.27, 43)	e, f
At least one foreign born parent	0.14	(0.08, 0.20)	0.13	(0.09, 0.16)	0.14	(0.09, 0.18)	0.17	(0.11, 0.23)	
Child attended daycare before age 3 years	0.59	(0.50, 0.68)	0.68	(0.63, 0.73)	0.62	(0.55, 0.68)	0.59	(0.50, 0.67)	e
Monthly household income									
€1200 or less	0.14	(0.12, 0.17)	0.17	(0.13, 0.19)	0.18	(0.13, 0.21)	0.19	(0.15, 0.22)	
€1201–€2000	0.39	(0.31, 0.43)	0.30	(0.25, 0.35)	0.30	(0.26, 0.34)	0.39	(0.32, 0.42)	
€2001–€3000	0.28	(0.25, 0.32)	0.32	(0.30, 0.35)	0.33	(0.28, 0.37)	0.30	(0.27, 0.36)	
€3001 or more	0.19	(0.15, 0.25)	0.21	(0.17, 0.26)	0.19	(0.14, 0.24)	0.12	(0.09, 0.19)	

95% confidence intervals are in parentheses

Child weight status was defined using World Health Organization child growth reference standards. Underweight defined as BMI-for-age-z-score < − 2. Normal weight defined as BMI-for-age-z-score ≥ − 2 and < 1. Overweight defined as BMI-for-age-z-score ≥ 1 and ≤ 2. Obese defined as BMI-for-age-z-score defined as ≥ 2

Mother and father BMI kept as continuous using self-reported height and weight to calculate BMI (kg/m^2^)

Good health was parent’s report of child’s overall health on a scale of good, fair, or poor

Daily screen time was parent reported if the child watched television or used a computer or video games daily

Mother with primary or less education was defined as whether the mother had completed primary school education or less

‘a’ indicates differences between underweight and normal weight; ‘b’ denotes significant differences between underweight and overweight; ‘c’ indicates differences between underweight and obese; ‘d’ denotes significant differences between normal and overweight; ‘e’ indicates significant differences between normal and obese; ‘f’ denotes differences between overweight and obese

All differences noted are significant at or below 5%

Missing values not shown

Data source: *Encuesta de Relaciones Inter e Intrageneracionales en la Infancia (2010)*

Children with underweight less often had an unemployed father compared to normal weight, children with overweight and children with obesity. They more often ate all 3 meals at home with family and less frequently engaged in daily physical activity.

Table 3 shows the results of multivariate multinomial logistic regression models, estimating the odds of being in an unhealthy weight category (underweight, overweight, and obesity) rather than normal weight.

**Table 3:** Odds of child’s unhealthy weight relative to normal weight children (5–10 years) and the family environment: results from unordered multinomial logistic regression using the nationally representative *Encuesta de Relaciones Inter e Intrageneracionales en la Infancia (2010)* (n = 844)

	Underweight vs. normal weight		Overweight vs. normal weight		Obesity vs. normal weight	
	OR	SD	OR	SD	OR	SD
Bio-demographics						
Boy	1.72*	0.23	1.65**	0.19	2.97***	0.22
Age (years)	0.97	0.06	1.03	0.06	0.90^+^	0.07
Good health	0.97	0.26	0.94	0.45	0.62	0.47
Parent’s BMI						
Mother	0.92**	0.04	1.02	0.03	1.05	0.03
Father	0.93^+^	0.04	1.04	0.03	1.08**	0.04
Mother’s age at childbirth	1.04	0.03	1.02	0.02	1.04^+^	0.03
Household structure						
Married parents	0.75	0.43	0.73	0.34	0.85	0.41
Has at least one sibling	0.97	0.26	0.79	0.20	0.84	0.24
Co-resides with a grandparent	1.00	0.44	0.82	0.35	1.65	0.36
Family activities						
Family eats all 3 meals together	0.72	0.40	1.37	0.27	1.41	0.39
Plays sports/does physical activity daily	0.61*	0.15	0.69*	0.19	0.73	0.22
Daily screen time	1.00	0.29	1.04	0.23	0.88	0.27
Family socio-economic characteristics						
Unemployed mother	0.61*	0.27	1.11	0.24	0.85	0.25
Unemployed father	0.29*	0.57	0.88	0.35	1.55	0.36
Mother with primary or less education	1.13	0.31	0.96	0.23	1.62^+^	0.26
At least one foreign born parent	1.54	0.33	1.07	0.27	1.73^+^	0.30
Child attended daycare before age 3 years	0.60*	0.16	0.77	0.20	0.63*	0.23
Monthly household income (ref: €1200 or less)						
€1201–€2000	1.19	0.37	1.00	0.29	1.53	0.34
€2001–€3000	0.61	0.41	1.07	0.31	1.27	0.38
€3001 or more	0.61	0.45	1.13	0.36	1.16	0.44

Models controlled for missing value with dummy variable adjustments

Weight status was defined using World Health Organization child growth reference standards. Underweight defined as BMI-for-age-z-score < − 2. Normal weight defined as BMI-for-age-z-score ≥ − 2 and < 1. Overweight defined as BMI-for-age-z-score ≥ 1 and ≤ 2. Obese defined as BMI-for-age-z-score defined as ≥ 2

Good health was parent’s report of child’s overall health on a scale of good, fair or poor

Daily screen time was parent reported if the child watched television or used a computer or video games daily

Mother with primary or less education was defined as whether the mother had completed primary school education or less

*SD* standard deviations are in parentheses

^+^ p < 0.1; * p < 0.05; ** p < 0.001; *** p < 0.0001

Data source: *Encuesta de Relaciones Inter e Intrageneracionales en la Infancia (2010)*

Column 1 presents the odds of being in the underweight rather than the normal weight category. Boys had 72% higher odds of being underweight compared with girls. Risks of underweight decreased with father’s (0.93) and mother’s BMI (0.92). Children who were physically active daily were less likely to have underweight rather than normal weight (0.61), as were children with working parents (mother: 0.61; father: 0.29) and those who had attended daycare before the age of 3 years (0.60).

Column 2 shows the odds of overweight compared to normal weight. Boys were 65% more likely to have overweight than normal weight compared with girls; children who were physically active were less likely to have overweight than normal-weight (0.69).

Column 3 reports the odds of obesity compared to normal weight. Boys’ odds of obesity than normal-weight were almost 3 times higher than those of girls. Odds of obesity decreased with child’s age (0.90) but increased with the mother’s age at the child’s birth (1.04). Odds of having obesity rather than normal-weight increased by 8% with each additional BMI point of the father. Children of mothers with only primary education (1.62) and of foreign-born parents (1.73) were more likely to have obesity. Children who had attended day-care before age 3 years were substantially less likely to have obesity than those who had not (0.63).

This study examined the weight status of school-aged children ages 5–10 years in Spain using a national family survey. Less than half (46%) of children had healthy weight, with underweight, overweight and obesity contributing to this high level of unhealthy weight. Findings are consistent with documented increases in overweight and obesity in Europe [8, 40]. Findings also highlight high levels of underweight, perhaps associated with the increase in food insecurity associated with the economic crisis of the last decade (> 1 SD below the mean), normal weight, overweight (1 to < 2 SD above the mean), and obesity (> 2 SD above the mean) [41] and that still persists 10 years later [6].

The most consistent correlates of unhealthy weight among school-aged children were gender, parents’ BMI, daycare attendance, and socioeconomic status. Most notably, boys were more likely than girls to have underweight or obesity. The risk of both underweight and obesity increased with their parents’ weight, consistent with previous findings from Spain [23]. The school environment from a young age in Spain may be beneficial, consistent with another study from Spain reporting that children who ate lunch at school had lower chances of obesity [31]. Spanish schools typically have a 2-h lunch break consisting of a 30-min lunch followed by over an hour of recess.

The characteristics that were associated with lower risks of underweight but not with lower risks of obesity were limited physical activity and parents’ unemployment. Characteristics associated with lower risks of obesity but not with risks of underweight were being younger, having an older or less educated mother, and having a foreign-born parent, highlighting the importance of the family structure in the study of weight differences.

## Limitations

This study was limited by cross-sectional and parent-reported data. Parent reports of children’s height and weight are more easily collected than direct measures in large-scale studies, but self-reported and parent-reported data have been shown to be systematically biased [42], warranting caution in the interpretation of results. Yet, parents’ weight estimation is more accurate than other weight estimation methods [43].

This study evaluated the overarching concerns of unhealthy weight among children in Spain, rather than focusing solely on obesity [10]. The contextual data explored here provided a more detailed understanding of the family contexts of unhealthy weight than is possible with other European data [8]. Spain’s economic growth has taken over a decade to return to pre-2008 levels [44], and the patterns of unhealthy weight documented here in 2010 may be linked with financial distress [7, 32].

Our findings suggest that obesity was coupled with underweight among children in Spain. That underweight and obesity were largely predicted by the same characteristics is an important consideration, highlighting that both types of malnutrition may be tied with family resources. The coexistence of underweight and obesity in a western setting is an important consideration for policies; ensuring healthy activity patterns and nutritious calories for children are strategies that may address both types of malnutrition. Further research should consider if these patterns persist in times of economic wellbeing or whether obesity and underweight are explained by different sets of characteristics under different economic circumstances.

## Additional file

**Additional file 1.** *Encuesta de Relaciones Inter e Intrageneracionales en la Infancia 2010. Questionnaire for Children 5*–*10* *years.* This is the full questionnaire for families with children 5–10 used for data collection in Spanish.
