# Supplementary material for: Unhealthy weight among children in Spain and the role of the home environment
Source: BMC Res Notes. 2018 Aug 15;11:591. doi: 10.1186/s13104-018-3665-2 (PMC6094473; doi:10.1186/s13104-018-3665-2)
Supplement: Supplementary file 1 — Additional file 1. Encuesta de Relaciones Inter e Intrageneracionales en la Infancia 2010. Questionnaire for Children 5–10 years. This is the full questionnaire for families with children 5–10 used for data collection in Spanish. [file 13104_2018_3665_MOESM1_ESM.docx]

**Additional File 1**

**Encuesta de Relaciones Inter e Intrageneracionales en la Infancia 2010 Questionnaire for Children 5-10 years**

**CUESTIONARIO 2 (NIÑOS DE 5-10 AÑOS)**

Buenos días estamos realizando un estudio de la Universidad de Barcelona sobre cómo educan a sus hijos las familias. Si fuera tan amable, ¿podría dedicarnos 10 minutos a responder un cuestionario?

Datos sociodemográficos del hijo/a

1. ¿Vive algún niño/a de 0 a 10 años en esta casa?

-Sí

-No (FIN)

6. El menor que vive en su hogar ¿Es niño o niña?

-Varón

-Mujer

7. ¿En que mes y año nació el niño/a? (MES Y AÑO REFERENCIA FEBRERO 2010)

7.1 -Mes:

7.2 –Año:

-NS/NC (NO LEER)

2. ¿Es Vd. El padre/madre responsable del niño?

-Padre

-Madre

-Pareja del padre/ madrastra (FIN)

-Pareja de la madre/ padrastro (FIN)

-Tutor/a (FIN)

-Ninguno de los anteriores (FIN)

3. ¿Vive el menor habitualmente con usted? *Habitualmente significa que pasan en el mismo hogar 3 o más noches a la semana*

-Sí

-No (FIN)

4. Sexo del entrevistado. *Anotar sin preguntar.*

-Mujer

-Varón

5. Podría decirme si Vd está ….

-Trabajando

-Parado (no tiene trabajo pero está buscando)

-Inactivo (no busca empleo. Ama de casa, estudiante, baja permanente)

APLICAR EL CUESTIONARIO PERTINENTE.

CUESTIONARIO 1: SI HA NACIDO DESPUÉS DE 1 FEBERO DE 2005

CUESTIONARIO 2: SI HA NACIDO ANTES DE 1 FEBRERO DE 2005

8. Incluido usted, ¿cuántas personas viven en el hogar? (*Pasan en el mismo hogar 3 o más noches a la semana*)

-Número de personas: _______

-NS/NC (NO LEER)

9. ¿Qué otras personas, además de Vd. viven con el niño/a en este hogar? (MULTIPLES RESPUESTAS) Imputar madre o padre dependiendo del sexo del entrevistado

-Madre del niño/a

-Padre del niño/a

-Su pareja (que no es el padre o madre del niño/a)

-Abuelo/a del niño

-Hermano/a del niño

-Hermanastro/a del niño

-Otros familiares del niño.

-Otros no familiares del niño.

-Ninguno

-NS/NC (NO LEER)

(PREGUNTAR SÓLO SI UNO DE LOS PROGENITORES NO VIVE CON EL MENOR)

10. Veo que el padre/madre no vive en el mismo hogar. ¿Me podría decir el motivo? (Leer opciones)

-Estamos divorciados/separados. Tenemos custodia compartida (*pasar a pregunta 11*)

-Estamos divorciados/separados. Yo tengo la custodia. (*pasar a pregunta 11*)

-Estamos divorciados/separados. Mi expareja tiene la custodia. (*pasar a pregunta 11*)

-El padre/madre ha fallecido *(pasar a pregunta 14)*

-El padre/madre nunca ha reconocido al niño/a *(pasar a pregunta 14)*

-Otros motivos. Especificar____________ *(pasar a pregunta 14)*

-NS/NC (NO LEER)

***FILTRO1***

1. ***Si P9(1|2) | P10(1|2|3)***
2. ***Resto***

***FILTRO2***

1. ***Si P9(1|2) | P10(1|2|3|4)***
2. ***Resto***

(FILTRO P.10 = 1 O P.10 =2 P.10=3)

11. ¿Con qué frecuencia su hijo/a ve al padre/madre con el que no reside habitualmente?

-Diariamente o casi cada día

-Al menos una vez por semana

-Al menos una vez cada quince días

-Al menos una vez al mes

-Nunca/casi nunca

-NS/NC (NO LEER)

(FILTRO P.10 = 1 O P.10 =2 P.10=3)

12. ¿Recibe o paga Vd. una cantidad económica a su pareja?

-Sí, recibo

-Sí, pago

-Ni recibo, ni pago *(pasar a pregunta 14)*

-NS/NC (NO LEER) *(pasar a pregunta 14)*

FILTRO P12(1|2)

13. ¿Con qué regularidad recibe/paga la cantidad acordada? Recibe/paga de/a su ex pareja la cantidad de forma…

-Regular (según lo acordado)

-Irregular (algunas veces sí, en otras se producen retrasos o impagos)

-Nunca

-NS/NC (NO LEER)

(A TODOS)

Ahora vamos a hablar del cuidado de su hijo/a.

14. ¿Me podría decir cuál es el nivel de implicación que usted tiene en la vida del niño/a? Está… *(Por nivel de implicación entendemos el tiempo que pasa con el niño/a, cuida de él/ella, se preocupa por sus necesidades, le presta atención etc.)*

-Muy implicado

-Bastante Implicado

-Poco implicado

-Nada implicado

-NS/NC (NO LEER)

FILTRO FILTRO1(1)

15. ¿Y el padre/madre del niño/a?

-Muy implicado

-Bastante Implicado

-Poco implicado

-Nada implicado

-NS/NC (NO LEER)

(A TODOS)

16. ¿En el último mes ha recibido alguna ayuda de alguien que no viva habitualmente en el hogar para cuidar al niño/a?

- Sí

- No (IR A PREGUNTA 17)

- NS/NC (NO LEER)

FILTRO P16(1)

16b. ¿De quién? (ESPONTÁNEA)

- Pareja/ Cónyuge

- Excónyuge/expareja

- Madre/padre/ Suegro/a

- Hermano/a/Cuñado

- Mi Hijo/a mayor

- Otros familiares

- Amigo/a o vecino/a

- Canguro

- Otras personas no familiares

- NC/ NS (NO LEER)

FILTRO P16(1)

16c. ¿Con qué frecuencia?

-Cada día o casi diariamente

-Alguna vez

-Nunca/casi nunca

-NS/NC (NO LEER)

(A TODOS)

17. En la última semana, ¿Me podría decir con qué frecuencia usted o alguna persona que vive en su hogar ha realizado alguna de las siguientes actividades con el niño/a?

SI CONTESTA “DIARIAMENTE” O “ALGUNA VEZ” PREGUNTAR 17b

17b. ¿Quién realiza esas actividades con el niño/a más a menudo?

| En la última semana, usted o alguna persona que vive en su hogar… | ¿Con qué frecuencia? | | | ¿Quién lo hace más a menudo? | | | |
| --- | --- | --- | --- | --- | --- | --- | --- |
|  | Diariamente/  Casi a diario | Alguna vez | Ninguna vez | Vd. | Su pareja | Los dos por igual | Otra persona |
| a. le lee o cuenta cuentos |  |  |  |  |  |  | |
| b. Hace manualidades con él/ella (pinta, hace artesanía, plastilina) |  |  |  |  |  |  | |
| c. Le han llevado a visitar a unos familiares |  |  |  |  |  |  | |
| d. le ha llevado a pasear o al parque |  |  |  |  |  |  | |
| e. Le ha abrazado, besado, o hecho cosquillas… |  |  |  |  |  |  | |

FILTRO: PREGUNTAR P. 17c SI CONTESTA “DIARIAMENTE” O “ALGUNA VEZ” A LA PREGUNTA P17.1a (le lee o cuenta cuentos) Y TIENE DE 5 A 7 AÑOS DE EDAD

17c. Cuando Vd. o alguien que vive en su hogar lee al niño/a, dígame con qué frecuencia realiza las siguientes actividades

|  | A menudo | Alguna vez | Nunca |
| --- | --- | --- | --- |
| Paran de leer y le preguntan al niño/a qué ve en las ilustraciones |  |  |  |
| Paran de leer y señalan letras |  |  |  |
| Piden al niño/a que lea con usted |  |  |  |
| Hablan de cosas que ocurren en el cuento cuando terminan de leerlo |  |  |  |

(A TODOS)

18. En una semana normal ¿Con qué frecuencia la familia entera se reúne para desayunar, comer o cenar? …(EN CASO DE FAMILIA MONOPARENTAL NO SE PREGUNTA POR EL PADRE/MADRE QUE NO VIVE HABITUALMENTE CON EL NIÑO/A)

|  | Cada día/ Casi cada día | Alguna vez por semana | Nunca | NS/NC (NO LEER) |
| --- | --- | --- | --- | --- |
| Desayunar |  |  |  |  |
| Comer |  |  |  |  |
| Cenar |  |  |  |  |

19. ¿Considera que el tiempo en general que Vd. pasa con su hijo/a es…?

- Más que suficiente

- Suficiente

- No suficiente

- NC/ NS (NO LEER)

FILTRO FILTRO1(1)

20. ¿Y el que pasa el padre/madre con su hijo/a…?

- Más que suficiente

- Suficiente

- No suficiente

- NC/ NS (NO LEER)

21. ¿En una semana normal su hijo/a…con qué frecuencia realiza las siguientes actividades?

SI CONTESTA “CADA DÍA” O “ALGUNA VEZ” PREGUTNAR 21b

21b ¿Con quién realiza esas actividades más a menudo?

|  | ¿Con qué frecuencia? | | |  | ¿Con quién realiza esa actividad más a menudo? | | | | |
| --- | --- | --- | --- | --- | --- | --- | --- | --- | --- |
|  | Cada día o casi cada día | Alguna vez | Ninguna vez |  | Solo/a | Con Vd. o su pareja | Hermanos/as | Otros  familiares | Amigos |
| Juega a la consola, ordenador o juegos similares |  |  |  |  |  |  |  |  |  |
| Practica algún deporte o actividad física |  |  |  |  |  |  |  |  |  |
| Va al cine, teatro o museo |  |  |  |  |  |  |  |  |  |
| Mira la televisión o videos |  |  |  |  |  |  |  |  |  |
| Toca un instrumento |  |  |  |  |  |  |  |  |  |
| Va a visitar a otros familiares |  |  |  |  |  |  |  |  |  |

22. ¿Cuantos días a la semana su hijo/a lleva amigos/as a casa?

0 1 2 3 4 5 6 7

-NS/NC (NO LEER)

23. Normalmente, cuando su hijo/a y sus amigos/as pasan tiempo juntos/as (ELEGIR UNA DE LAS TRES)

-…hay algún adulto en la misma habitación con ellos?

-…hay algún adulto en la misma casa pero en una habitación distinta?

-…no hay ningún adulto en casa?

- NS/NC (NO LEER)

24. ¿Cuántos amigos de su hijo/a diría Vd. que conoce?

-Todos o la mayoría

-Algunos

-Casi ninguno/ ninguno

-NS/NC (NO LEER)

25. ¿Y a cuántos de los padres de los amigos/as de su hijo/a conoce?

-Todos o a la mayoría

-Algunos

-A casi ninguno/ ninguno

-NS/NC (NO LEER)

A continuación le vamos a hacer una serie de preguntas acerca de cómo es su hijo/a y cómo se comporta

26. ¿En qué medida está de acuerdo con las siguientes afirmaciones respecto a su hijo/a?

|  | Muy de acuerdo | Bastante de acuerdo | En desacuerdo | NS/NC (NO LEER) |
| --- | --- | --- | --- | --- |
| Le gusta pasar tiempo con otras personas |  |  |  |  |
| Se mete en conflictos o peleas |  |  |  |  |
| Le gusta molestar a otros |  |  |  |  |
| Le gusta reír |  |  |  |  |
| A veces está triste |  |  |  |  |
| A veces se siente solo/a |  |  |  |  |
| Normalmente está de buen humor |  |  |  |  |
| Pierde los papeles fácilmente |  |  |  |  |
| No puede parar quieto/a |  |  |  |  |
| Es tímido/a |  |  |  |  |
| Le gusta probar cosas nuevas |  |  |  |  |
| A veces se asusta de las cosas o la gente |  |  |  |  |
| Se muestra enfadado/a con los demás con frecuencia |  |  |  |  |

27. A veces los niños/as se portan bastante bien y otras veces no. Le voy a leer una serie de formas de tratar a su hijo/a. ¿Podría decirme con qué frecuencia ha hecho lo siguiente en la última semana?

|  | Diariamente | Casi cada día | Alguna vez | Ninguna vez | NS/NC (NO LEER) |
| --- | --- | --- | --- | --- | --- |
| Felicita al niño/a porque hace las cosas bien |  |  |  |  |  |
| Le levanta la voz, o le grita |  |  |  |  |  |
| Le da un tiempo para que reflexione sobre lo que ha hecho |  |  |  |  |  |
| Le amenaza con castigarle |  |  |  |  |  |
| Le castiga (sin salir de la habitación, sin ver la tele, sin jugar al ordenador o videoconsola, etc.) |  |  |  |  |  |
| Le da un cachete |  |  |  |  |  |

28. Me podría decir con qué frecuencia se producen discusiones o tensiones en su casa por los siguientes motivos

|  | A menudo | Alguna vez | Nunca | NS/NC |
| --- | --- | --- | --- | --- |
| Hay tensión por el reparto de tareas domésticas |  |  |  |  |
| Hay tensión por el cuidado del niño/a |  |  |  |  |
| Hay tensión por dificultades económicas |  |  |  |  |
| Hay tensión por no disponer de tiempo personal para relajarse o desconectar |  |  |  |  |
| Hay tensión por el estrés en el trabajo (suyo o de su pareja) |  |  |  |  |

A continuación le vamos a realizar algunas preguntas acerca de la educación de su hijo/a y de cómo le va en la escuela

29. ¿Estuvo su hijo/a en la guardería en las siguientes edades?

|  | Sí | No | NS/NC |
| --- | --- | --- | --- |
| De 0 y 6 meses |  |  |  |
| Entre 7 meses y 1año |  |  |  |
| Con 1 año |  |  |  |
| Con 2 años |  |  |  |

(Si todas las P29 fueron “NO” no hacer P30)

30. ¿En general, estaba satisfecho/a con los siguientes aspectos respecto a la guardería?

|  | Muy satisfecho | Bastante | Poco | Nada | NS/NC |
| --- | --- | --- | --- | --- | --- |
| Número de monitores por aula |  |  |  |  |  |
| Preparación de los monitores |  |  |  |  |  |
| Horarios |  |  |  |  |  |

FILTRO: PREGUNTAR P31, P32, P33, P34 A PARTIR DE LOS 7 AÑOS

31. ¿En la última semana con qué frecuencia su hijo/a ha tenido que hacer deberes de la escuela?

-Cada día

-Casi cada día

-Alguna vez por semana

-Ninguna vez

NS/NC (NO LEER)

32. Considera que para su hijo/a hacer los deberes es una tarea…

-Fácil

-Ni fácil ni difícil

-Difícil

-NS/NC (NO LEER)

33. ¿Cuanto tiempo diría que dedican Vd y su pareja a la semana a hablar con su hijo/a sobre los deberes, ayudándole a hacerlos, repasándolos…? (SE TRATA DE LA SUMA DEL TIEMPO QUE DEDICA EL ENTREVISTADO Y SU PAREJA)

-Menos de una hora

-Entre una hora y tres horas

-Más de tres horas

-NS/NC (NO LEER)

34. ¿En su casa, el niño/a dispone de un lugar tranquilo para hacer los deberes?

-Sí

-No

-NS/NC (NO LEER)

(A TODOS)

35. ¿Está satisfecho/a con los resultados escolares de su hijo/a?

-Muy satisfecho/a

-Bastante satisfecho/a

-Poco satisfecho/a

-Nada satisfecho/a

-NS/NC (NO LEER)

36. Algunos niños/as tienen problemas en la escuela. El tutor o maestro de la escuela le ha comentado que su hijo/a…

|  | Si | No | NS/NC |
| --- | --- | --- | --- |
| Se muestra muy nervioso/a cuando tiene que intervenir en clase |  |  |  |
| Tiene problemas de concentración/ prestar atención |  |  |  |
| Tiene problemas para seguir alguna materia o asignatura |  |  |  |

37. Y su hijo/a, ¿le ha comentado que….

|  | Si | No | NS/NC |
| --- | --- | --- | --- |
| Le tiene miedo a su profesor/a |  |  |  |
| Le da miedo algún compañero/a |  |  |  |
| No le gusta estudiar |  |  |  |

38. ¿En el último año ha utilizado alguno de los siguientes servicios o instalaciones?

|  | Sí | No | NS/NC |
| --- | --- | --- | --- |
| Servicios de apoyo a las familias (espacios familiares, escuela de padres…) |  |  |  |
| Ayudas económicas a familias, o becas de las administraciones públicas |  |  |  |
| Apoyo de un asistente o trabajador social |  |  |  |
| Psicólogo escolar, pedagogo |  |  |  |
| Actividades extraescolares (actividades artísticas, clases de refuerzo, actividades deportivas, etc.) |  |  |  |

SI CONTESTA AFIRMATIVAMENTE A UTILIZACIÓN DE ACTIVIDADES EXTRAESCOLARES preguntar 39, 39b, 40, 41

39. ¿Asiste su hijo/a a alguna de las siguientes actividades extraescolares?

SI CONTESTA AFIRMATIVAMENTE PREGUNTAR P39b

39b. ¿Con qué frecuencia asiste habitualmente durante la semana?

|  |  |  | ¿Con qué frecuencia asiste habitualmente a la semana? | | | |
| --- | --- | --- | --- | --- | --- | --- |
|  | SÍ | NO | Tres o más veces a la semana | 2 veces a la semana | 1 vez a la semana | Ninguna vez |
| Actividades artísticas (danza, arte, música…) |  |  |  |  |  |  |
| Clases de refuerzo |  |  |  |  |  |  |
| Clases de idiomas (inglés, francés, etc.) |  |  |  |  |  |  |
| Actividades deportivas |  |  |  |  |  |  |
| Actividades escolares que requieren participación del padre o madre |  |  |  |  |  |  |
| Otras actividades que no haya mencionado |  |  |  |  |  |  |

40. Le voy a leer una serie de motivos por los que la gente lleva a sus hijos a una actividad extraescolar. Dígame si en su decisión influyeron mucho, bastante, poco o nada las siguientes razones.

|  | Mucho | bastante | Poco | Nada | NS/NC |
| --- | --- | --- | --- | --- | --- |
| Los horarios del trabajo suyos o de su pareja |  |  |  |  |  |
| La posibilidad de que su hijo pudiera pasar tiempo con amigos/as y compañeros/as |  |  |  |  |  |
| En estas actividades dispone de materiales y recursos de los que no dispone en casa |  |  |  |  |  |
| Porque enseñan a los niños a ser disciplinados |  |  |  |  |  |
| Para estimular aptitudes e intereses de su hijo/a |  |  |  |  |  |
| Porque se lo pidió su hijo/a |  |  |  |  |  |

41. En general ¿A su hijo/a le gusta ir al/los programa(s)/actividad(es) extraescolar(es) que realiza?

Mucho

Bastante

Poco

Nada

NS/NC

(A TODOS)

42. Diría que su hijo/a en general se siente feliz siempre, a menudo, algunas veces, nunca o casi nunca en las siguientes situaciones…

|  | Siempre | A menudo | Algunas veces | Nunca o casi nunca | NS/NC (NO LEER) |
| --- | --- | --- | --- | --- | --- |
| Con su familia |  |  |  |  |  |
| Con sus amigos/as |  |  |  |  |  |
| En el barrio |  |  |  |  |  |
| En la escuela |  |  |  |  |  |
| En general |  |  |  |  |  |

43. ¿En el último mes cuantos días ha faltado su hijo/a a clase por motivos de salud? _________días

-NS/NC (NO LEER)

44. ¿Cómo evaluaría, en general, la salud de su hijo/a?

-Buena (IR A PREGUNTA 45)

-Regular

-Mala

-NS/NC (NO LEER)

FILTRO P44(2|3|101|102)

44b. ¿Su hijo/a tiene alguna discapacidad física o mental diagnosticada por un médico?

-Sí

-No

-NS/NC (NO LEER)

(A TODOS)

45. ¿Me podría decir aproximadamente el peso del niño/a?

________Kg.

-NS/NC (NO LEER)

46. ¿Y su altura?

________cm.

-NS/NC (NO LEER)

**Características socioeconómicas de los padres**

47. ¿Me podría decir en que año nació Vd.?

Año ________

-NS/NC (NO LEER)

FILTRO FILTRO1(1)

48. ¿Y el padre/madre del niño/a?

Año__________

-NS/NC (NO LEER)

(A TODOS)

49. ¿Me podría decir su estado civil?

Casado/a

Soltero/a

Separado

Divorciado

Viudo

Pareja de hecho

-NS/NC (NO LEER)

*En caso de que esté casado/a…P49(1)*

49b. ¿Está casado/a con el padre/madre del niño/a?

-Sí

-No

-NS/NC (NO LEER)

*Si es pareja de hecho P49(6)*

49c. ¿Se ha registrado como pareja de hecho en un ayuntamiento o registro oficial?

- Sí

- No

-NS/NC (NO LEER)

(A TODOS)

50. ¿El lugar de nacimiento de Vd. Es España?

-Sí

-No

-NS/NC (NO LEER)

*En caso negativo…*

50b. ¿En qué país nació?

_______________país

-NS/NC (NO LEER)

50c. ¿Desde cuando vive en España?

50c_1__________años

50c_2___________meses

-NS/NC (NO LEER)

FILTRO FILTRO2(1)

51. ¿El lugar de nacimiento de su pareja es España?

-Sí

-No

-NS/NC (NO LEER)

*En caso negativo…*

51b. ¿En qué país nació?

_______________país

-NS/NC (NO LEER)

51c. ¿Desde cuando vive en España?

51c_1__________años

51c_2___________meses

-NS/NC (NO LEER)

52. ¿Me podría decir aproximadamente cuánto pesa Vd.?

_________Kg.

-NS/NC (NO LEER)

53. Y ¿me podría especificar su altura?

_______cm

-NS/NC (NO LEER)

FILTRO FILTRO1(1)

54. Y el padre/madre del niño/a ¿Cuánto pesa aproximadamente?

_______ Kg.

-NS/NC (NO LEER)

FILTRO FILTRO1(1)

55. ¿Su altura?

_______ cm.

-NS/NC (NO LEER)

56. ¿Me podría decir cuál es su nivel estudios que acabados?

-Sin estudios/Estudios Primarios (EGB, Bachillerato elemental, Graduado escolar)

-Secundaria (ESO, Bachillerato, BUP, COU)

-FP1/ Ciclos Formativos de Grado Medio

-FP2 / Ciclos Formativos de Grado Superior

-Diplomatura Universitaria, Arquitectura Técnica o Ingeniería Técnica

-Licenciatura Universitaria, Arquitectura Superior o Ingeniería Superior

-Doctorado, Estudios de Postgrado, Máster, MIR o similar

-Otros

-NS/NC (NO LEER)

FILTRO FILTRO1(1)

57. ¿Me podría decir cuál es el nivel estudios acabados por el padre/madre del menor?

-Sin estudios/Estudios Primarios (EGB, Bachillerato elemental, Graduado escolar)

-Secundaria (ESO, Bachillerato, BUP, COU)

-FP1/ Ciclos Formativos de Grado Medio

-FP2 / Ciclos Formativos de Grado Superior

-Diplomatura Universitaria, Arquitectura Técnica o Ingeniería Técnica

-Licenciatura Universitaria, Arquitectura Superior o Ingeniería Superior

-Doctorado, Estudios de Postgrado, Máster, MIR o similar

-Otros

-NS/NC (NO LEER)

*En caso de que el entrevistado/a esté ocupado/a…(P5=1)*

58. ¿Qué tipo de contrato tiene usted?

-Asalariado/a con contrato indefinido

-Asalariado/a con contrato temporal

-Asalariado/a sin contrato

-Autónomo/a o profesional sin empleados

-Empresario/a con empleados

59. ¿Trabaja a tiempo completo o a tiempo parcial?

-Tiempo parcial (Menos de 35h)

-Tiempo completo (35h o más)

FILTRO P9(1|2|3)

60. ¿Cuál es la situación laboral de su pareja?

-Trabaja

-Está Parado/a (IR A PREGUNTA P61)

-No trabaja (IR A PREGUNTA P61)

-No tengo pareja (IR A PREGUNTA P61)

-NS/NC (NO LEER)

FILTRO P60(1)

60a. ¿Qué tipo de contrato tiene?

-Asalariado/a con contrato indefinido

-Asalariado/a con contrato temporal

-Asalariado/a sin contrato

-Autónomo/a o profesional sin empleados

-Empresario/a o profesional con empleados

-NS/NC (NO LEER)

FILTRO P60(1)

60b. ¿Trabaja a tiempo completo o a tiempo parcial?

-Tiempo parcial (Menos de 35h)

-Tiempo completo (35h o más)

-NS/NC (NO LEER)

(A TODOS)

61. Una familia como la suya, ¿cuánto dinero cree que necesita aproximadamente para llegar a final de mes?

________euros

-NS/NC (NO LEER)

62. Actualmente, entre todos los miembros del hogar (incluido el entrevistado) y por todos los conceptos (prestaciones públicas o privadas, ayudas, otros ingresos como alquileres, acciones, etc.) ¿de cuántos ingresos netos disponen por término medio en su hogar al mes?

- Menos de 600 euros al mes

- De 600 a 1200 euros al mes

- De 1201 a 2000 euros al mes

- De 2001 a 3000 euros al mes

- De 3001 a 5000 euros al mes

- De 5001 a 7000 euros al mes

- Más de 7000 euros al mes

- NS/NC (NO LEER)

MUCHAS GRACIAS
